# Supplementary material for: RNF167 activates mTORC1 and promotes tumorigenesis by targeting CASTOR1 for ubiquitination and degradation
Source: Nat Commun. 2021 Feb 16;12:1055. doi: 10.1038/s41467-021-21206-3 (PMC7887217; doi:10.1038/s41467-021-21206-3)
Supplement: Supplementary file 1 — Supplementary Information [file 41467_2021_21206_MOESM1_ESM.pdf]

Supplementary Information

for

RNF167 activates mTORC1 and promotes tumorigenesis by targeting CASTOR1 for ubiquitination and degradation

Tingting Li[1], Xian Wang[1], Enguo Ju[1], Suzane Ramos da Silva[1], Luping Chen[1], Xinquan Zhang[1], Shan Wei[1], Shou-Jiang Gao[1]*

[1]UPMC Hillman Cancer Center, Department of Microbiology and Molecular Genetics, University of Pittsburgh, Pittsburgh, PA15213, USA

* Correspondence: gaos8@upmc.edu

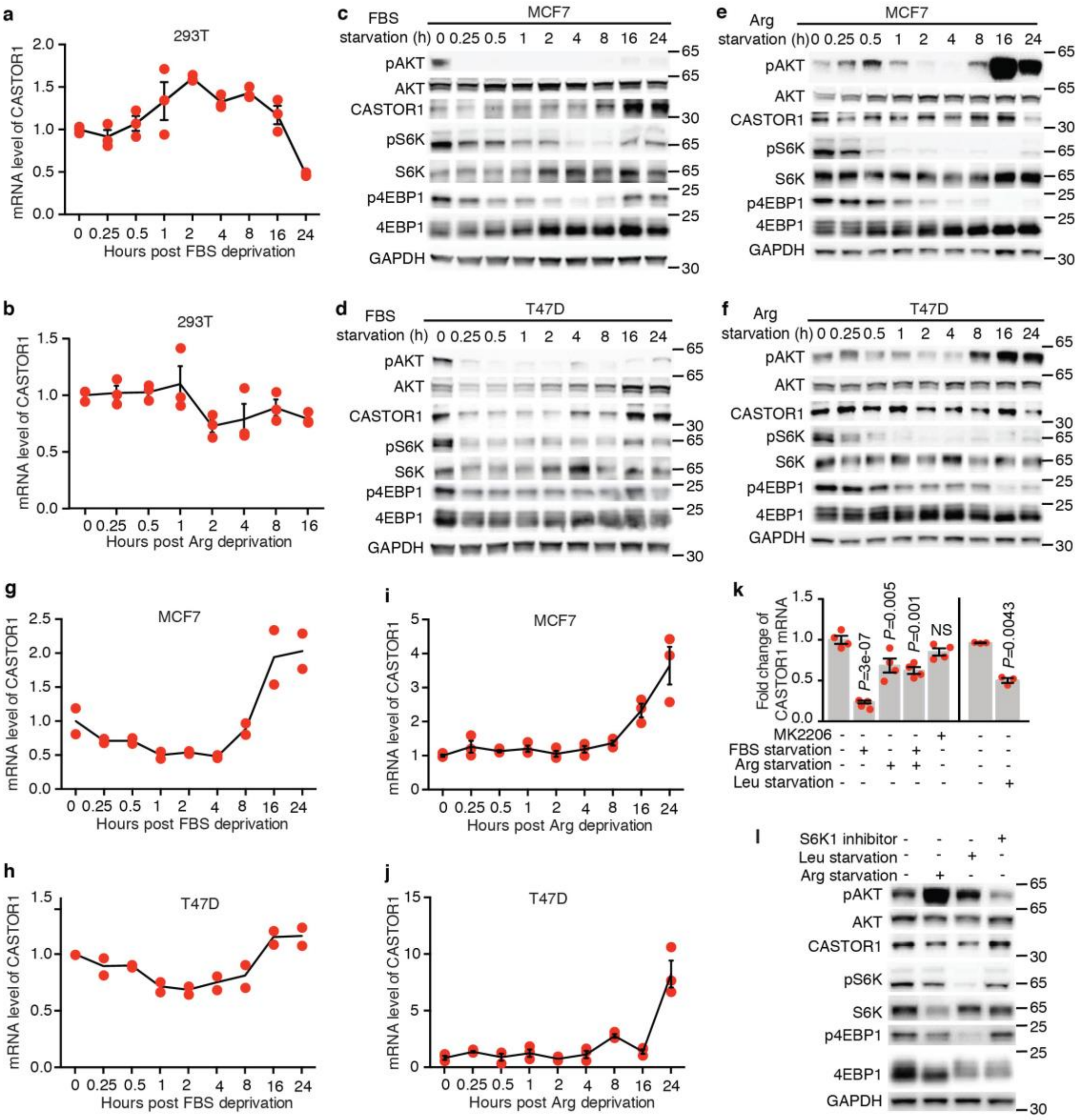

Supplementary Figure 1 CASTOR1 regulation by nutrients and growth factors in multiple cell lines. a, b CASTOR1 mRNA level was slightly decreased by FBS (**a**) and arginine (**b**) deprivation in 293T cells; data are presented as mean values ± SEM (n=3 independent experiments). **c, d** Kinetics of CASTOR1 protein level, and activation status of AKT and mTORC1 following FBS deprivation in MCF7 (**c**) and T47D (**d**) cells. FBS deprivation, particularly for more than 16 h, increased CASTOR1 protein level. **e, f** Kinetics of CASTOR1 protein level, and activation status of AKT and mTORC1 following arginine deprivation in MCF7 (**e**) and T47D (**f**) cells. **g, h** Kinetics of CASTOR1 mRNA level following FBS deprivation in MCF7 (**g**) and T47D (**h**) cells; data are presented as mean values and the actual data points from n=2 independent experiments. **i, j** Kinetics of CASTOR1 mRNA level following arginine deprivation in MCF7 (**i**) and T47D (**j**) cells; data are presented as mean values ± SEM (n=3 independent experiments). **k** CASTOR1 mRNA level following

deprivation of FBS, arginine or leucine, or AKT inhibition. FBS, arginine or leucine deprivation but not AKT inhibition decreased endogenous CASTOR1 mRNA level in 293T cells. Data are presented as mean values ± SEM and *P* values were calculated by one-way ANOVA followed by Tukey post-hoc test (n=4 independent experiments) except for leucine deprivation results, which are presented as mean values ± SEM and *P* values were calculated by two-sided unpaired Student t test (n=3 independent experiments); NS, not significant. **l** arginine or leucine deprivation and S6K inhibitor inactivated mTORC1 but only arginine or leucine deprivation rather than S6K inhibitor activated AKT and reduced CASTOR1 protein level. Blots in **c**-**f** and **l** are representatives of n=3 independent experiments. Source data are provided in Source Data file.

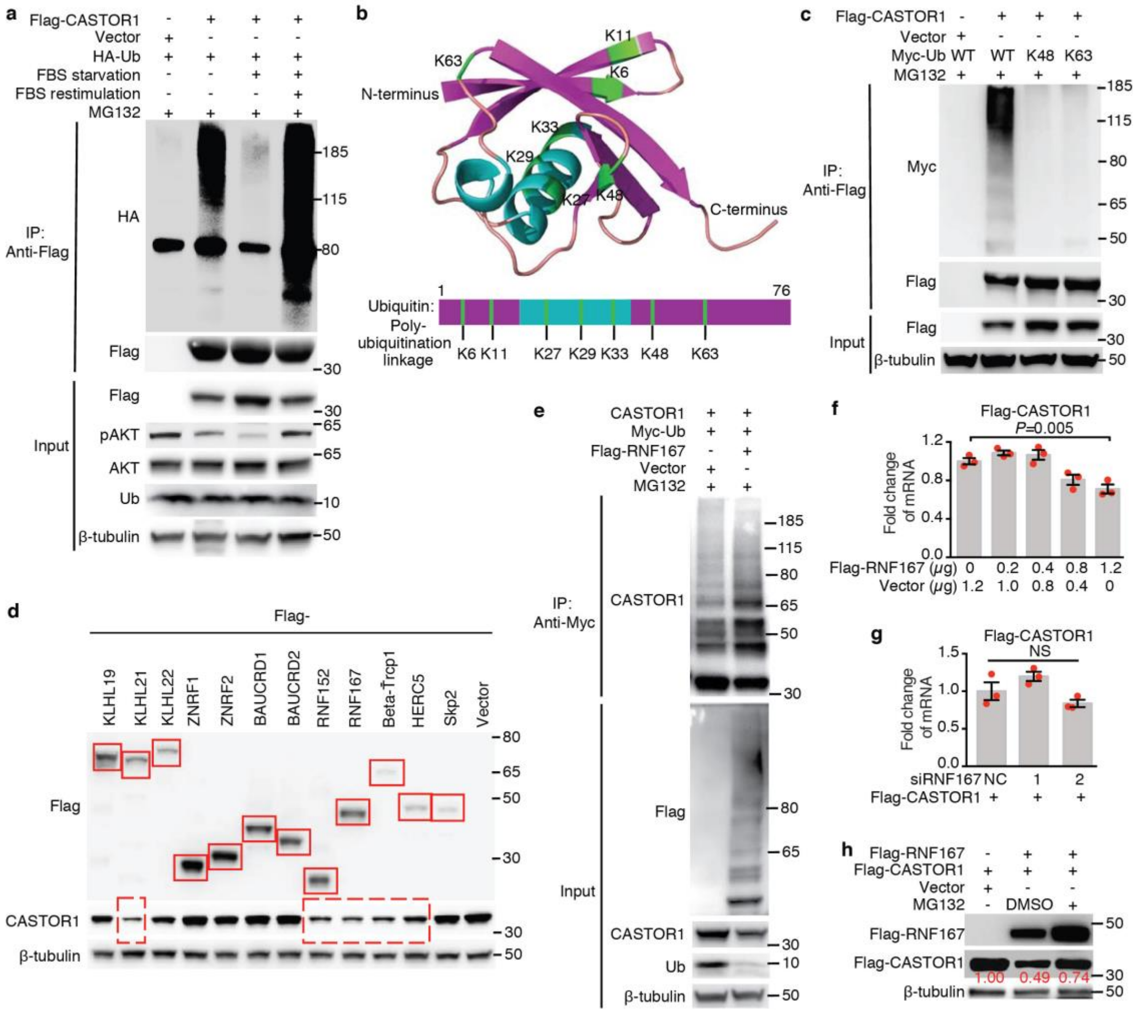

Supplementary Figure 2 RNF167 mediates K29-linked polyubiquitination and degradation of CASTOR1 in response to growth factors. a FBS starvation decreased while re-stimulation restored CASTOR1 ubiquitination. **b** A schematic illustration of ubiquitin structure and the seven lysine residues in ubiquitin responsible for polyubiquitination linkage. The structure of ubiquitin is adopted from: http://www1.rcsb.org/structure/1ubq. **c** CASTOR1 was not tagged by K48- or K63-linked polyubiquitination. **d** Screening of E3 ligases that regulated CASTOR1 protein level. **e** RNF167 overexpression increased CASTOR1 ubiquitination. **f, g** RNF167 overexpression had no effect on CASTOR1 mRNA level except at higher doses (>0.8 µg), which showed a marginal reduction (**f**), while RNF167 knockdown had no effect on CASTOR1 mRNA level (**g**). Data are presented as mean values ± SEM and *P* values were calculated by one-way ANOVA followed by Tukey post-hoc test (n=3 independent experiments); NS, not significant. **h** MG132 partially rescued RNF167-mediated CASTOR1 downregulation. Blots in **a**, **c-e** and **h** are representative of n=3 biological independent experiments. Source data are provided in Source Data file.

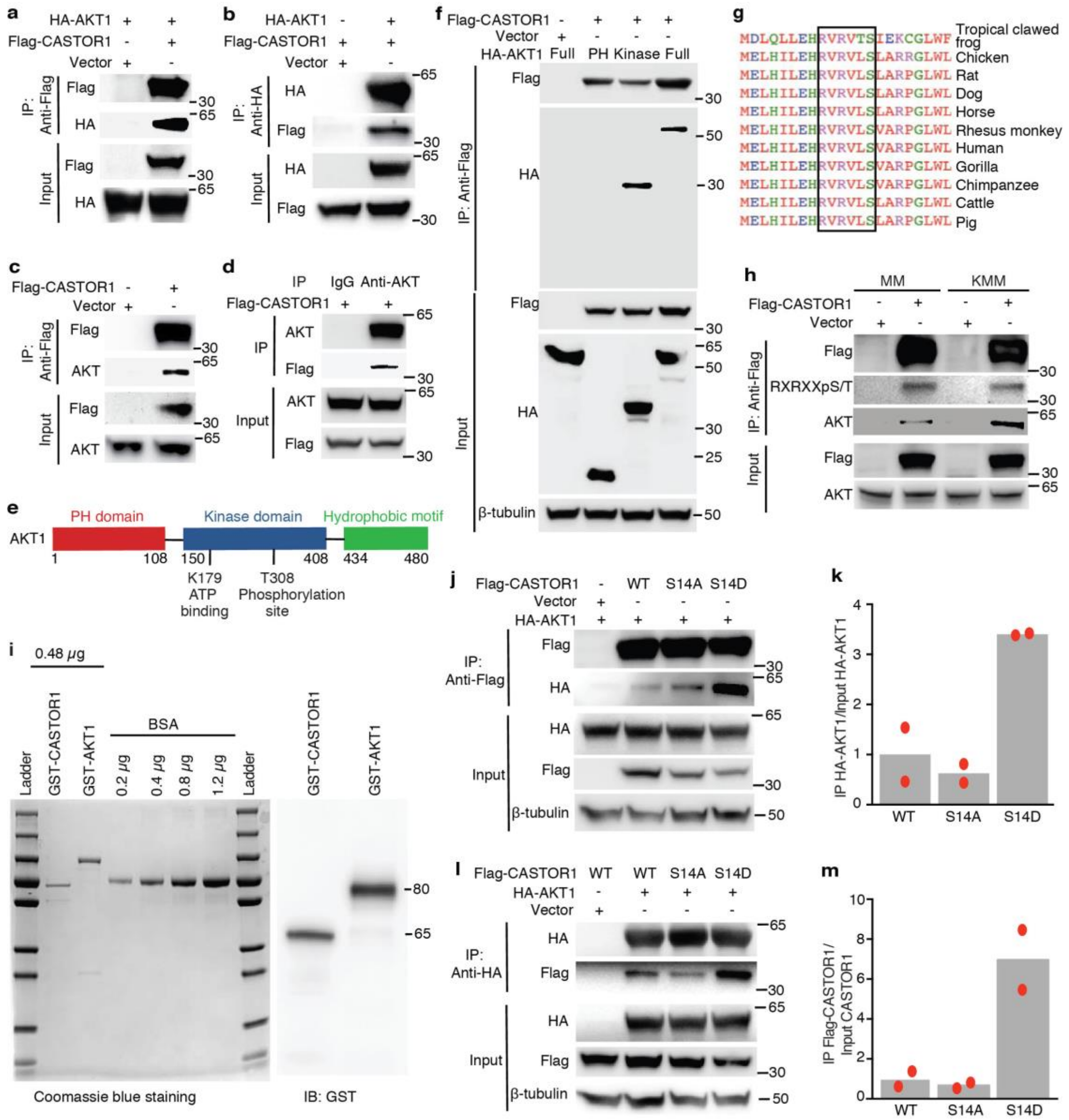

Supplementary Figure 3 AKT1 interacts with and phosphorylates CASTOR1 at S14. a-d CASTOR1 interacted with exogenous (**a, c**) and endogenous AKT (**b, d**). **e** Schematic illustration depicting AKT1 domains consisting of PH and kinase domains, and a hydrophobic motif. K179 is the ATP-binding site and T308 is the phosphorylation site required for AKT kinase functionality. **f** CASTOR1 interacted with the AKT1 kinase domain. **g** AKT phosphorylation consensus motif in CASTOR1 was conserved among vertebrates. Alignment of the CASTOR1 protein sequence was performed using the MUSCLE algorithm[29]. **h** AKT interacted with and phosphorylated CASTOR1 in rat cells. **i** Coomassie blue staining and immunoblotting analysis with an anti-GST antibody to examine the purity of recombinant GST-AKT1 and GST-CASTOR1 proteins. **j-m** CASTOR1 S14D had stronger binding to AKT1 than WT and S14A had (**j** and **l**); results presented as mean values and the actual data points from n=2 independent experiments (**k** and **m**). Blots in **a-d**, **f**,

h-j and **l** are representatives of n=3 independent experiments. Source data are provided in Source Data file.

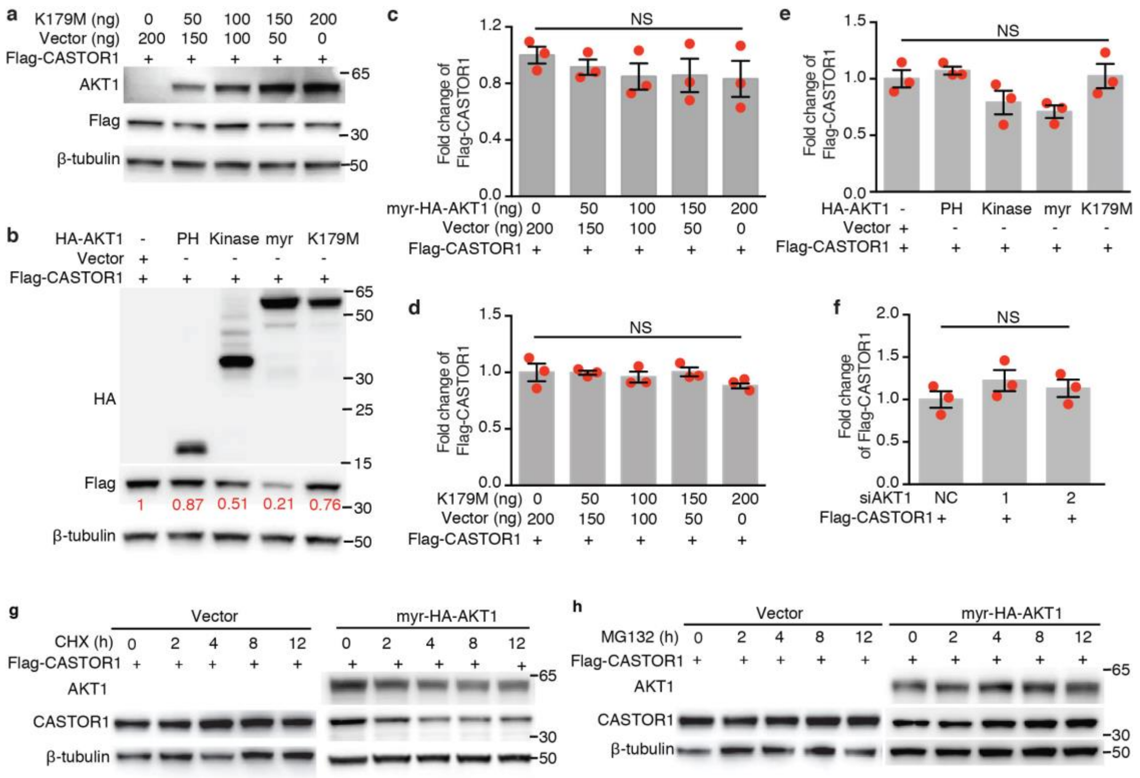

Supplementary Figure 4 AKT1-mediated phosphorylation of CASTOR1 promotes its proteasome-dependent degradation. a AKT1 kinase dead mutant K179M did not affect CASTOR1 protein level. **b** AKT1 kinase domain was sufficient to decrease CASTOR1 protein level but to a lesser extent than the AKT1 WT. **c-f** myr-HA-AKT1, AKT1-K179M, different AKT1 domains and different AKT1 siRNAs (siAKT1s) did not affect CASTOR1 mRNA level. Data are presented as mean values ± SEM and *P* values were calculated by one-way ANOVA followed by Tukey post-hoc test (n=3 independent experiments); NS, not significant. **g, h** AKT1 accelerated CASTOR1 degradation. 293T cells co-transfected with Flag-CASTOR1 and myr-HA-AKT1 for 36 h were treated with either cycloheximide (CHX) (**g**) or MG132 (**h**). Blots in **a**, **b**, **g** and **h** are representatives of n=3 independent experiments. Source data are provided in Source Data file.

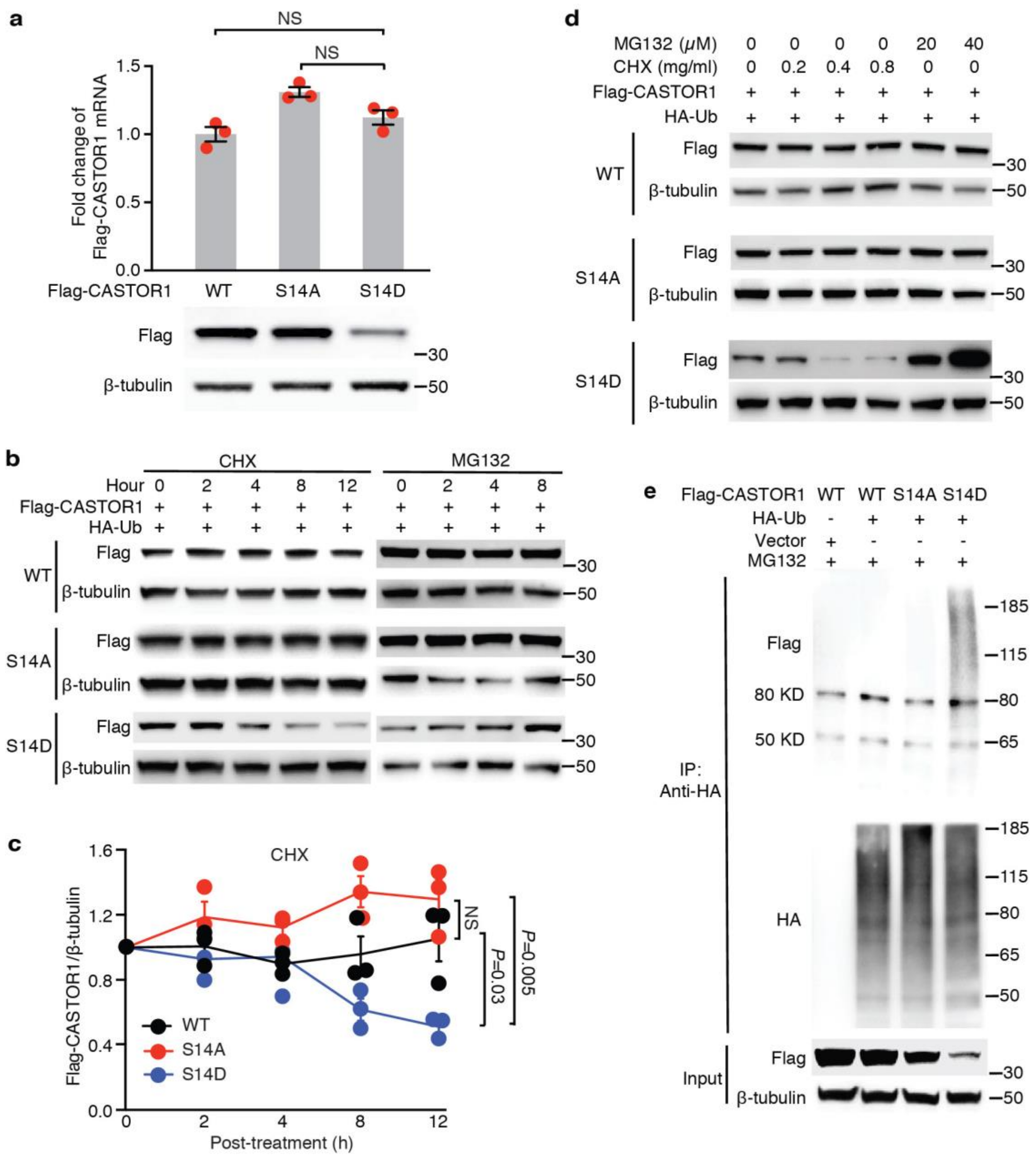

Supplementary Figure 5 AKT1-mediated phosphorylation of CASTOR1 promotes its proteasome-dependent degradation. a WT, S14A and S14D had similar mRNA levels while the protein level of S14D level was lower than those of WT and S14A. Data are presented as mean values ± SEM and *P* values were calculated by one-way ANOVA followed by Tukey post-hoc test (n=3 independent experiments); NS, not significant. **b, c** CASTOR1 S14D had faster turnover than WT and S14A had. The protein level of CASTOR1 WT, S14A or S14D was examined following treatment with either CHX or MG132 for the indicated times (**b**), and the relative levels were quantified and presented in (**c**). For panel **c**, data are presented as mean values ± SEM and *P* values were calculated by one-way ANOVA followed by Tukey post-hoc test (n=3 independent experiments); NS, not significant. **d** S14D had a lower protein

level following treatment with cycloheximide (CHX) but a higher protein level following treatment with MG132 than WT and S14A had. Treatment with CHX or MG132 was carried out for 12 h with the indicated concentrations. **e** CASTOR1 S14D had an increased ubiquitination level compared to WT and S14A. Blots in **a**, **b** and **d** are representatives of n=3 independent experiments, and blots in **e** are representatives of n=2 independent experiments. Source data are provided in Source Data file.

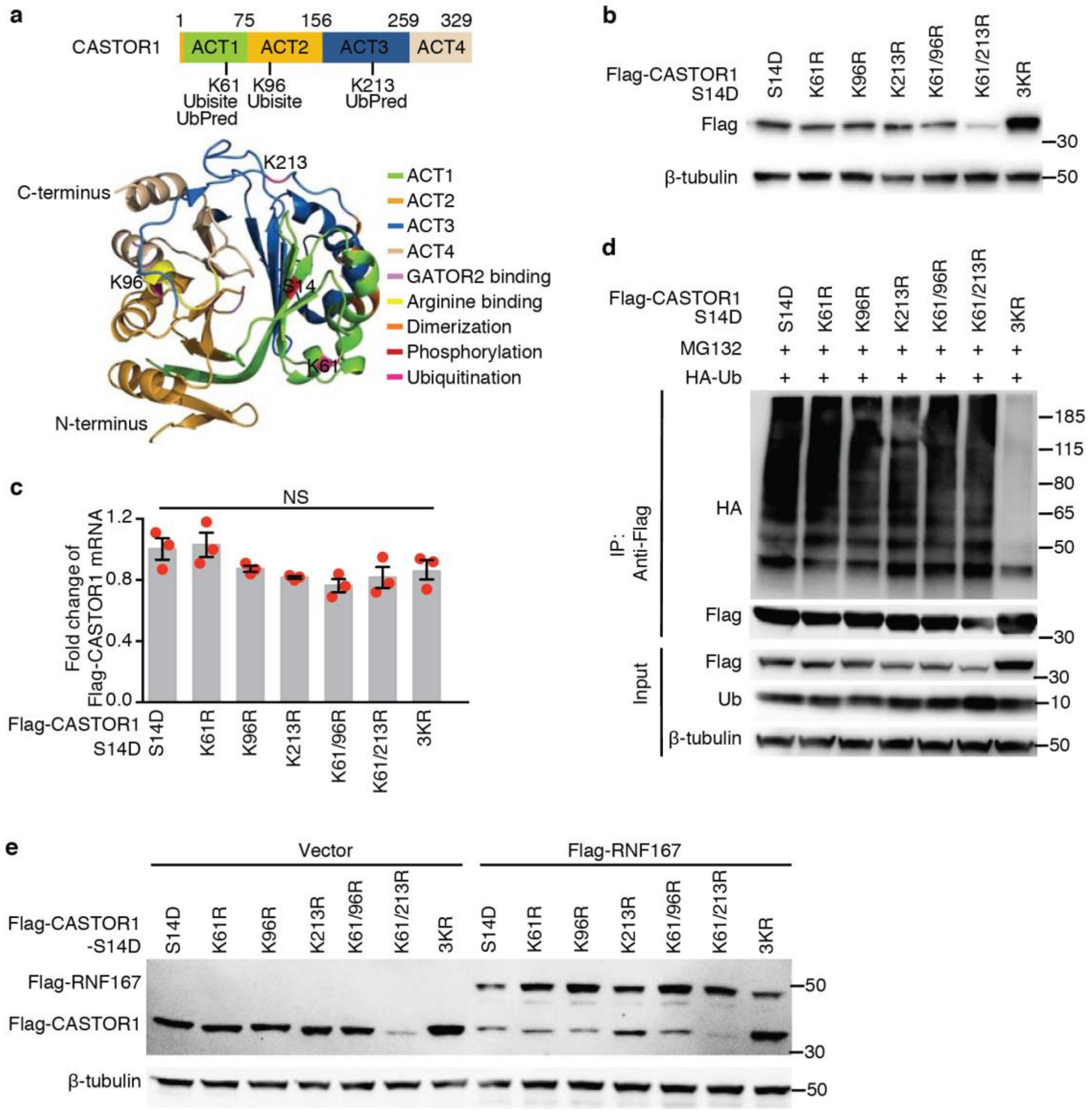

Supplementary Figure 6 CASTOR1 is marked by K29-linked polyubiquitination at K61, K96 and K213. a A schematic illustration of CASTOR1 structure and the three putative ubiquitination lysine residues responsible for polyubiquitination. The structure of CASTOR1 is adopted from: http://www1.rcsb.org/structure/5I2C. **b, c** Simultaneous mutations of CASTOR1 lysines K61, K96 and K213 to arginine (3KR) were required to stabilize the protein (**b**) but had no effect on mRNA level (**c**); results shown are presented as means ± SEM from n=3 independent experiments (**c**), and one-way ANOVA followed by Tukey post-hoc test was used for the statistical analysis. NS, not significant. **d, e** The K61, K96 and K213 triple mutant 3KR of Flag-CASTOR1 S14D was resistant to RNF167-mediated ubiquitination (**d**) and degradation (**e**). Blots in **b**, **d** and **e** are representatives of n=3 independent experiments. Source data are provided in Source Data file.

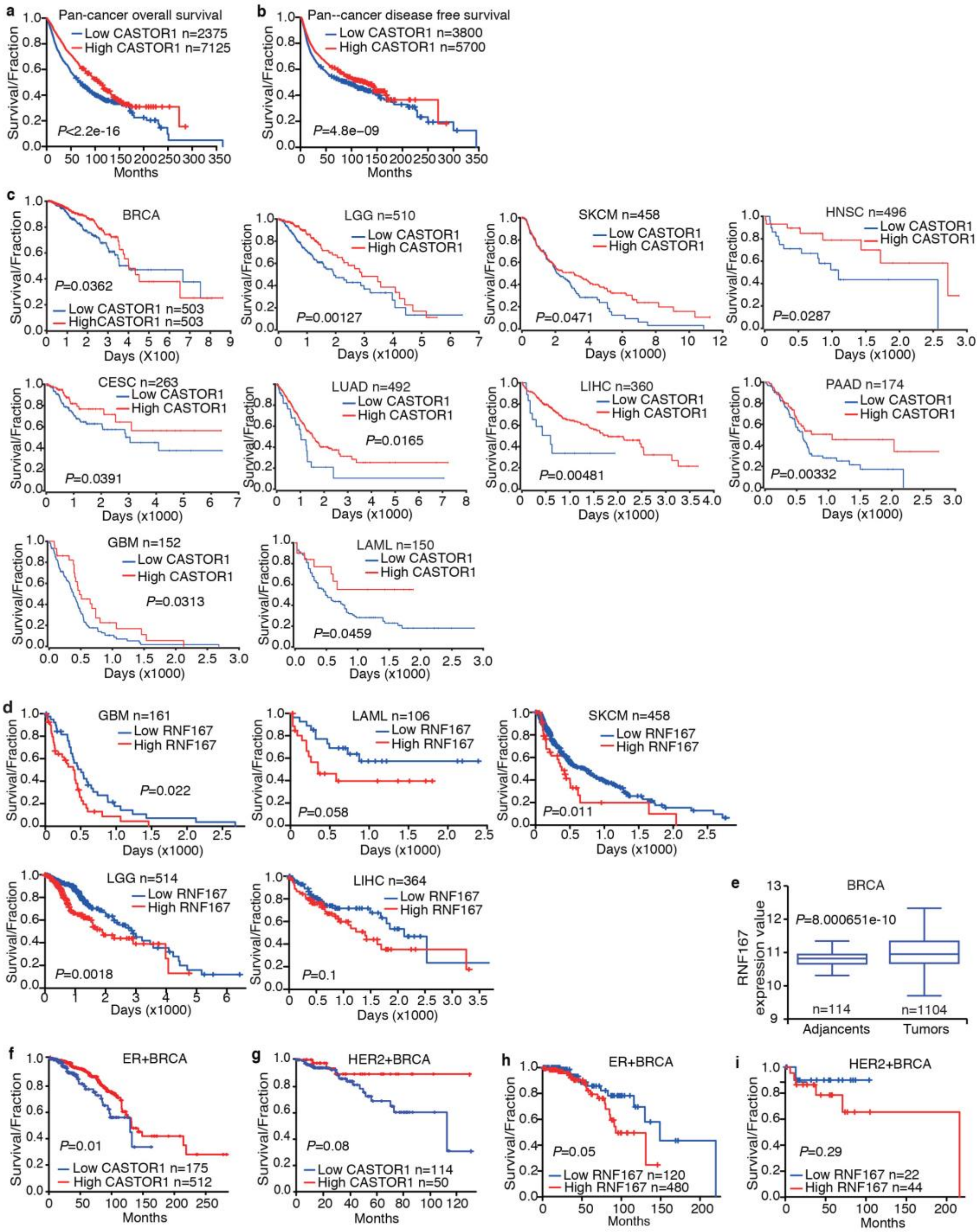

Supplementary Figure 7 Expression levels of CASTOR1 and RNF167 regulate mTORC1 and predict cancer survival in different types of cancer. **a, b** A lower CASTOR1 expression level was associated with poor overall survival (**a**) and disease-free survival (**b**) in pan-cancer analysis. **c** A lower CASTOR1 mRNA expression level was associated with poor survival in 10 types of cancer including breast invasive carcinoma (BRCA), brain lower grade glioma (LGG), skin cutaneous melanoma (SKCM), head and neck squamous cell carcinoma (HNSC), cervical squamous cell carcinoma and endocervical adenocarcinoma (CESC), lung adenocarcinoma (LUAD), liver hepatocellular

carcinoma (LIHC), pancreatic adenocarcinoma (PAAD), glioblastoma multiforme (GBM) and acute myeloid leukemia (LAML). **d** A high RNF167 mRNA expression level was associated with poor survival in 5 types of cancer including GBM, LAML, SKCM, LGG and LIHC. **e** The RNF167 expression level was higher in breast cancer tumors (n=1,104) than the adjacent normal tissues (n=114). The boundary closest to the zero indicates the 25th percentile, a line within the box means the median, and the boundary of the box farthest from zero marks the 75th percentile. Whiskers (error bras) above and below the box indicate the minima and maxima. **f, g** A lower CASTOR1 expression level was associated with poor survival of specific breast cancer subtypes including HER2-positive (HER2+) (**f**) and ER positive (ER+) (**g**) subtypes. **h, i,** A high RNF167 expression level was associated with poor survival of ER+ (**h**) and HER2+ (**i**) subtypes of breast cancer. Analyses were performed with the TCGA database (**a-i**). Panels **a-d** and **f-i**) were analyzed by two-sided Log-rank test.

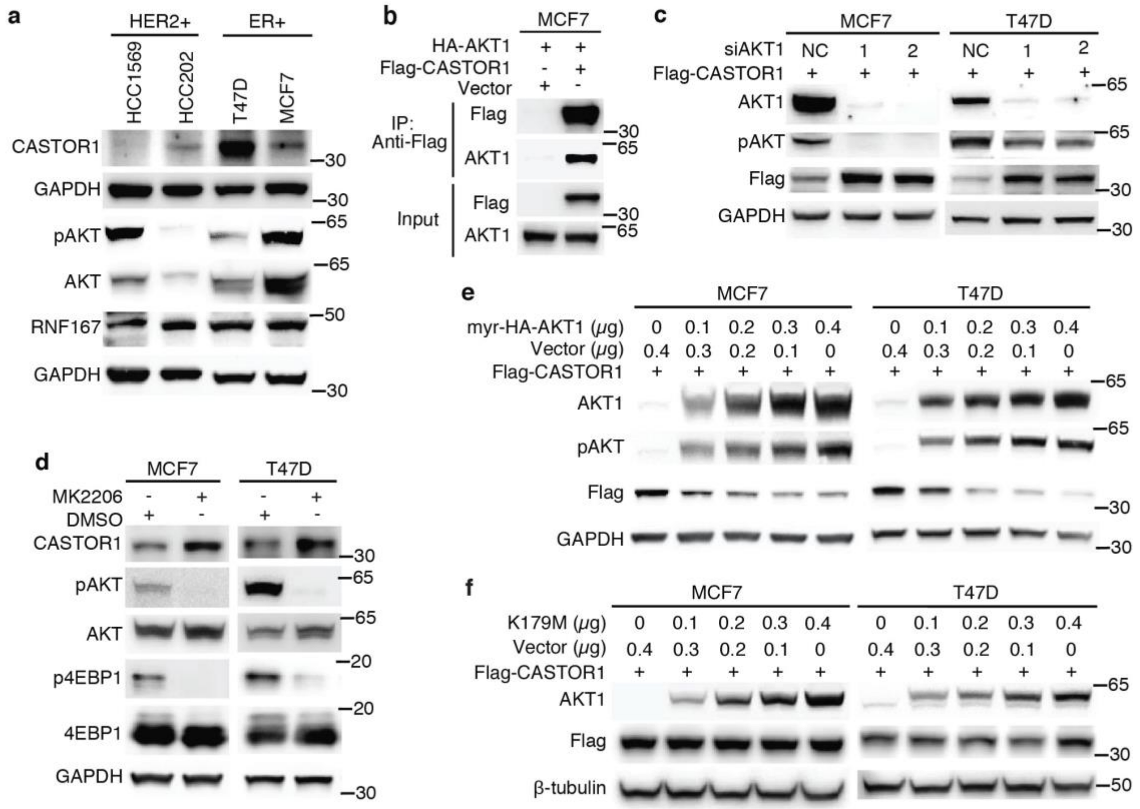

Supplementary Figure 8 AKT1-mediated phosphorylation and degradation of CASTOR1 in breast cancer cells. a CASTOR1 protein level was negatively correlated with AKT activation in ER+ and HER2+ breast cells, respectively. **b** CASTOR1 interacted with AKT1 in MCF7 cells. **c** AKT1 silencing increased CASTOR1 protein level in ER+ breast cancer cells. **d** AKT inhibitor MK2206 increased the endogenous CASTOR1 protein level in ER+ breast cancer cells. **e, f** overexpression of myr-HA-AKT1 (**e**) but not the AKT kinase dead mutant K179M (**f**) in MCF7 and T47D cells resulted in a dose-dependent reduction in CASTOR1 protein level. Blots in **a-f** are representatives of n=3 independent experiments**.** Source data are provided in Source Data file.

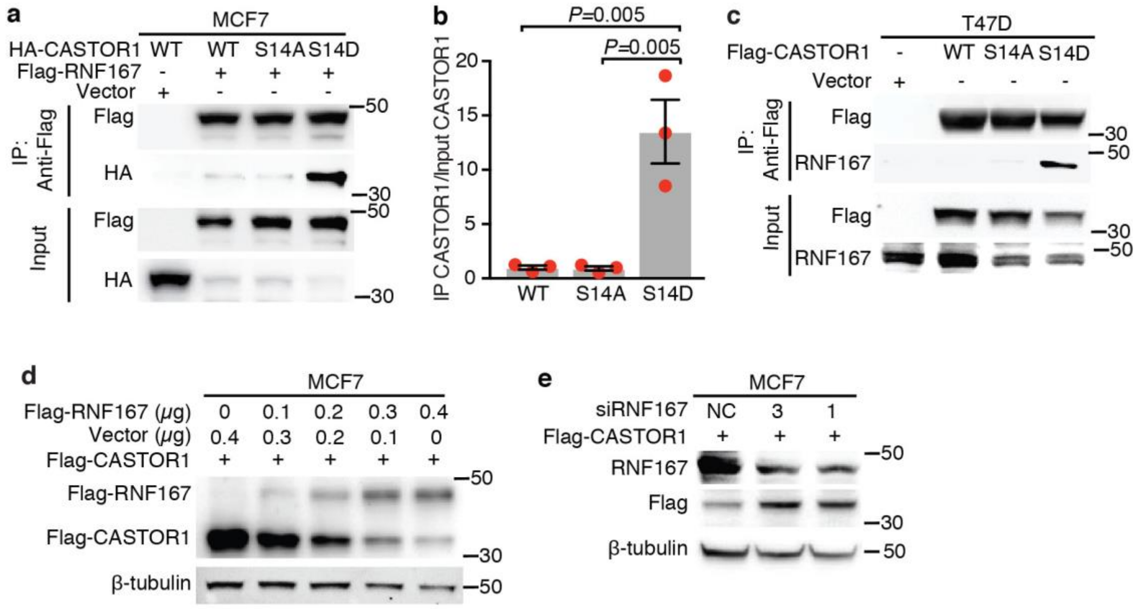

Supplementary Figure 9 AKT phosphorylation of CASTOR1 promoted RNF167-mediated CASTOR1 degradation in breast cancer cells. a-c CASTOR1 S14D had higher affinity to RNF167 than WT and S14A had in MCF7 cells (**a, b**) and T47D (**c**). For panel **b**, data are presented as mean values ± SEM and *P* values were calculated by one-way ANOVA followed by Tukey post-hoc test (n=3 independent experiments). **d, e** RNF167 overexpression (**d**) decreased while RNF167 knockdown (**e**) increased CASTOR1 expression in MCF7 cells. Blots in **a, c**-**e** are representatives of n=3 independent experiments. Source data are provided in Source Data file.

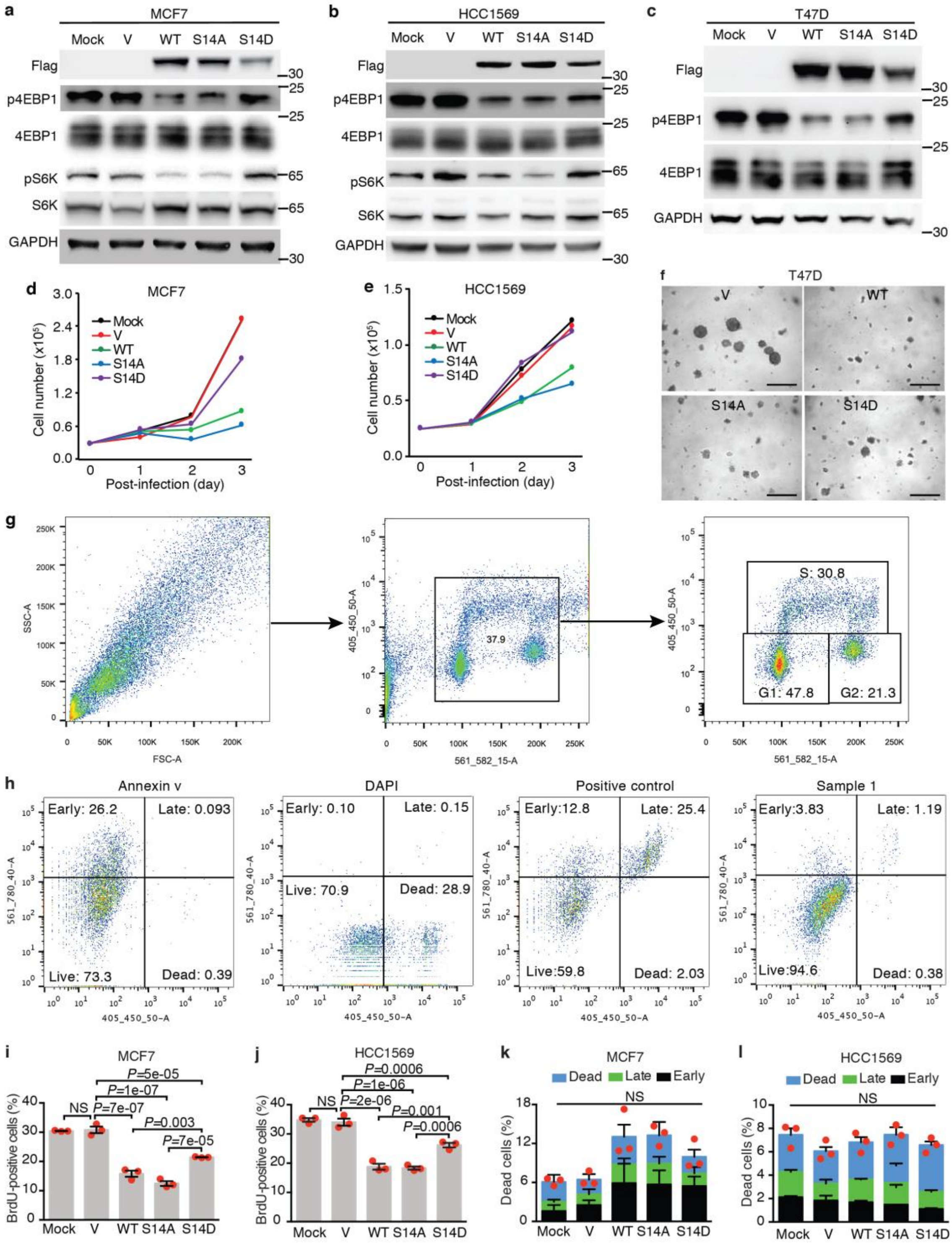

Supplementary Figure 10 CASTOR1 inhibits cell proliferation, cell cycle progression and colony formation in softagar of breast cancer cells by inactivating mTORC1. a-c Inhibition of mTORC1 activation was weaker by Flag-CASTOR1 S14D than WT and S14A in MCF7 (**a**), HCC1569 cells (**b**) and T47D (**c**). **d-f** CASTOR1 S14D had a weaker effect than WT and S14A had on suppressing cell proliferation in MCF cells (**d**) and HCC1569 cells (**e**), and colony formation in softagar of T47D cells (**f**); data in panels **d** and **e** are

representatives from n=3 independent experiments. Scale bars: 200 µM (**f**). **g** FACS sorting strategy used for BrdU analysis shown in panels **i** and **j**. **h** FACS sorting strategy used for apoptosis analysis shown in panels **k** and **l**. **i, j** Overexpression of Flag-CASTOR1 S14D induced weaker cell cycle arrest than WT and S14A did in ER+ MCF7 (**i**) and HER2+ HCC1569 (**j**). **k, l** Overexpression of Flag-CASTOR1 WT, S14A or S14D had minimal effect on apoptosis in ER+ MCF7 cells (**k**) and HER2+ HCC1569 cells (**l**). For panel **i-l,** data are presented as mean values ± SEM and *P* values were calculated by one-way ANOVA followed by Tukey post-hoc test (n=3 independent experiments); NS, not significant. Blots in **a**-**c** and image in **f** are representatives of n=3 independent experiments. Source data are provided in Source Data file.

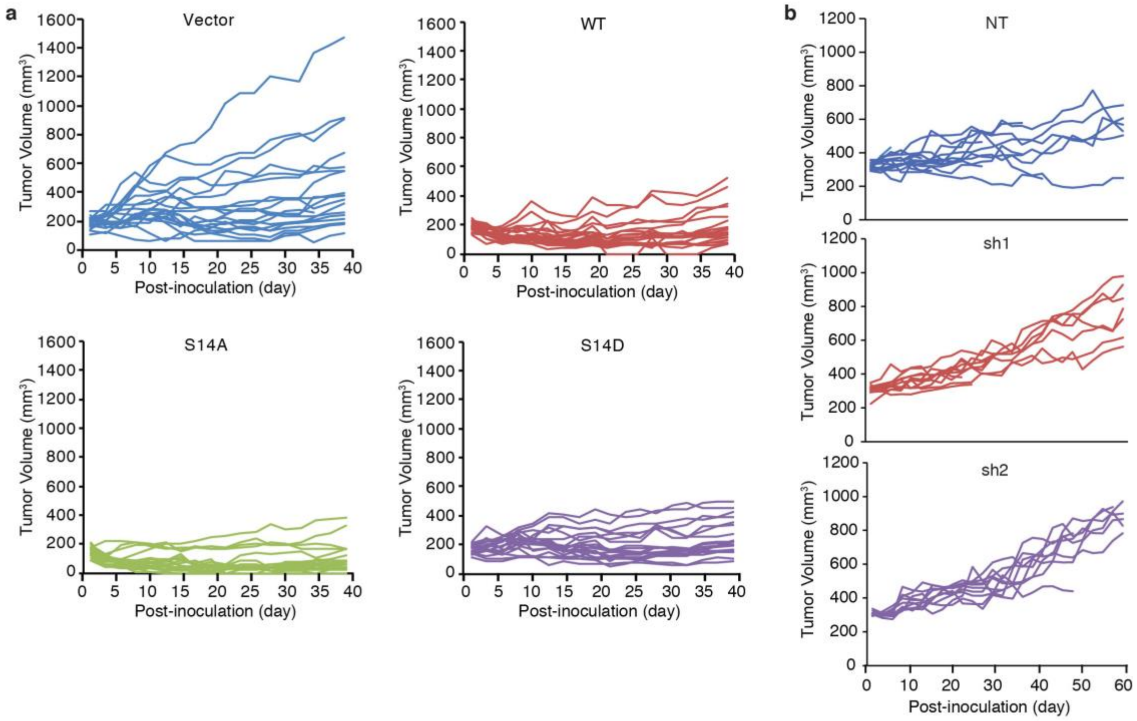

Supplementary Figure 11 AKT1-mediated phosphorylation and degradation as well as silencing of CASTOR1 promote breast cancer progression. a Individual tumor growth curves showing that CASTOR1 WT and S14A had more inhibitory effect on tumor growth than S14D had. **b** Individual tumor growth curve after CASTOR1 silencing revealed that CASTOR1 deletion promoted tumor growth.

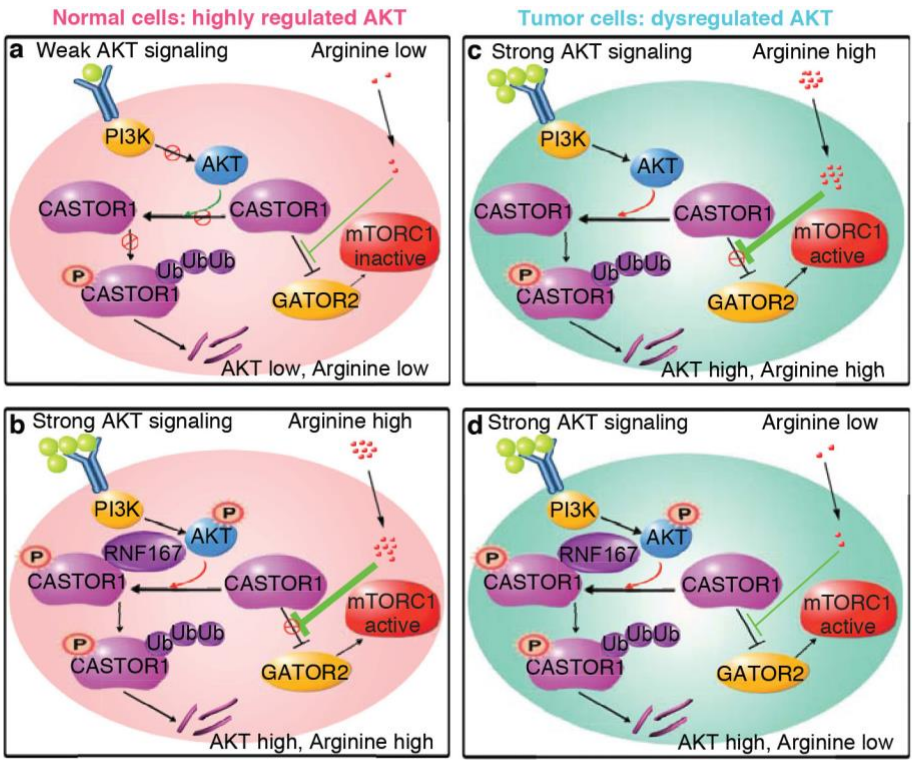

Supplementary Figure 12 Proposed model of AKT-mediated phosphorylation, and RNF167-dependent ubiquitination and degradation of CASTOR1, and mTORC1 activation in normal and cancer cells. a, b mTORC1 activity is highly regulated in response to growth factors and nutrients in normal cells. In quiescent or slow growing cells, cells are exposed to low levels of nutrients and growth factors. AKT is not or only weakly activated, and CASTOR1 is dephosphorylated and stabilized to sequester GATOR2 complex, leading to mTORC1 inactivation and slow growth or arrest of the cells (**a**). In fast growing cells, cells are exposed to high levels of growth factors and nutrients. On one hand, AKT is highly activated, which induces CASTOR1 phosphorylation followed by RNF167-mediated CASTOR1 degradation as well as reduces binding to MIOS. On the other hand, high levels of growth factors also stimulate uptake of nutrients including arginine, which disrupts CASTOR1-GATOR2 interaction leading to mTORC1 activation and anabolic growth of the cells (**b**). **c, d** mTORC1 activity is dysregulated and constitutively active as a result of dysregulated AKT signaling in cancer cells. Dysregulated AKT signaling as a result of mutation in AKT or its upstream pathways of growth factors could increase cell uptake of nutrients including arginine and strong AKT activation. CASTOR1 function is suppressed by both AKT and arginine. Specifically, AKT-mediated CASTOR1 phosphorylation results in its increased binding to RNF167 and hence its degradation, as well as its reduced binding to MIOS while arginine also dissociates CASTOR1 from MIOS and the GATOR2 complex, a positive regulator of mTORC1, leading to robust mTORC1 activation and anabolic growth of cancer cells (**c**). In fast growing solid tumors,

cancer cells are often deprived of nutrients including arginine, which fail to inhibit CASTOR1's function. However, constitutive AKT activation would continue to induce CASTOR1 destruction and reduce its binding to MIOS leading to mTORC1 activation and anabolic growth of cancer cells (**d**).

Supplementary Table 1. Summary of PCR primers and shRNAs

Rat Flag-CASTOR1-forward	5′TATGCGGCCGCGCCACCATGGACTACAAAGAC GATGACGACAAGATGGAACTTCACATCCAGAGC3′
Rat Flag-CASTOR1-reverse	5′ATAGGATCCCTATGGATCTTTGGAAGCCAGG3′
Human CASTOR1-forward	5’TATGCGGCCGCGCCACCATGGAGCTGCACA TCCTAGAAC3'
Human CASTOR1-reverse	5'ATAGGATCCTCAGGAAGCCAGGCCTTCCT3'
Human HA-CASTOR1-forward	5'TATGCGGCCGCGCCACCATGTACCCATACGATGT TCCAGATTACGCTATGGAGCTGCACATCCTAGAAC3'
Human HA-CASTOR1-reverse	5'ATAGGATCCTCAGGAAGCCAGGCCTTCCT3'
Human Flag-CASTOR1-forward	5’TATGCGGCCGCGCCACCATGGACTACAAAGACG ATGACGACAAGATGGAGCTGCACATCCTAGAAC3’
Human Flag-CASTOR1-reverse	5’ATAGGATCCTCAGGAAGCCAGGCCTTCCT3'
Human Flag-CASTOR1-S14A-forward	5'GCGGGTGCTGGCTGTCGCCCGTC3'
Human Flag-CASTOR1-S14A-reverse	5'ACCCGGTGTTCTAGGATG3'
Human Flag-CASTOR1-S14D-forward	5'GCGGGTGCTGGATGTCGCCCGTC3'
Human Flag-CASTOR1-S14D-reverse	5'ACCCGGTGTTCTAGGATG3'
Human Flag-CASTOR1-K61R-forward	5'GGAGGGCTTTCGAGAGCTGCCCC3'
Human Flag-CASTOR1-K61R-reverse	5'TCGTCCACCATAAGCGTG3'
Human Flag-CASTOR1-K96R-forward	5'TGGGGTCACCCGGATCGCCCGTTCGG3'
Human Flag-CASTOR1-K96R-reverse	5'GCAGCCTGCACTGCCGCA3'
Human Flag-CASTOR1-K213R-forward	5'CAGCACCCCCCGGGAGGCAGCCT3'
Human Flag-CASTOR1-K213R-reverse	5'TGCGAGTAGAAGAGGACATCTATG3'
shRNA non-targeting (NT) control	5’TTGTACTACACAAAAGTACTG3’
Human CASTOR1-sh1	5’GGAGCTGCACATCCTAGAACA3’
Human CASTOR1-sh2	5’GCTTTGATGAATGTGGCATCG3’

Supplementary Table 2. Summary of siRNAs

siRNA negative control (NC)	Sigma	Cat#SIC001
Human AKT1 siRNA-1	Sigma	Cat#SASI_Hs01_00105954
Human AKT1 siRNA-2	Sigma	Cat#SASI_Hs01_00105953
Human RNF167 siRNA-1	Sigma	Cat#SASI_Hs01_00201491
Human RNF167 siRNA-2	Sigma	Cat#SASI_Hs01_00201493
Human TSC2 siRNA-1	Sigma	Cat#SASI_Hs01_00127335
Human TSC2 siRNA-2	Sigma	Cat# SASI_Hs01_00127336
