# Supplementary material for: RNF167 activates mTORC1 and promotes tumorigenesis by targeting CASTOR1 for ubiquitination and degradation
Source: Nat Commun. 2021 Feb 16;12:1055. doi: 10.1038/s41467-021-21206-3 (PMC7887217; doi:10.1038/s41467-021-21206-3)
Supplement: Supplementary file 3 — Reporting Summary [file 41467_2021_21206_MOESM3_ESM.pdf]

nature research

Corresponding author(s): Shou-Jiang Gao

Last updated by author(s): Dec 22, 2020

Reporting Summary

Nature Research wishes to improve the reproducibility of the work that we publish. This form provides structure for consistency and transparency in reporting. For further information on Nature Research policies, see our Editorial Policies and the Editorial Policy Checklist.

Statistics

For all statistical analyses, confirm that the following items are present in the figure legend, table legend, main text, or Methods section.

n/a	Confirmed	
☐	☒	The exact sample size (*n*) for each experimental group/condition, given as a discrete number and unit of measurement
☐	☒	A statement on whether measurements were taken from distinct samples or whether the same sample was measured repeatedly
☐	☒	The statistical test(s) used AND whether they are one- or two-sided<br>*Only common tests should be described solely by name; describe more complex techniques in the Methods section.*
☒	☐	A description of all covariates tested
☒	☐	A description of any assumptions or corrections, such as tests of normality and adjustment for multiple comparisons
☐	☒	A full description of the statistical parameters including central tendency (e.g. means) or other basic estimates (e.g. regression coefficient) AND variation (e.g. standard deviation) or associated estimates of uncertainty (e.g. confidence intervals)
☐	☒	For null hypothesis testing, the test statistic (e.g. *F*, *t*, *r*) with confidence intervals, effect sizes, degrees of freedom and *P* value noted<br>*Give P values as exact values whenever suitable.*
☒	☐	For Bayesian analysis, information on the choice of priors and Markov chain Monte Carlo settings
☒	☐	For hierarchical and complex designs, identification of the appropriate level for tests and full reporting of outcomes
☒	☐	Estimates of effect sizes (e.g. Cohen's *d*, Pearson's *r*), indicating how they were calculated

Our web collection on statistics for biologists contains articles on many of the points above.

Software and code

Policy information about availability of computer code

Data collection	To acquire microscopy data, IX83 inverted microscope (Olympus) was used.<br>To acquire Flow Cytometry data, BD LSRFortessa system was used.<br>To acquire WB images, ImageLab (v5.2.1) was used.<br>To acquire qPCR data, Biorad CFX Maestro 1.1 (v4.1.2433.1219) was used.
Data analysis	Flowjo vX.0.7<br>Graphpad Prism v6.0 and v8.0<br>ImageLab v5.2.1<br>Photoshop v21.0.2<br>Adobe Illustrator v24.0.1

For manuscripts utilizing custom algorithms or software that are central to the research but not yet described in published literature, software must be made available to editors and reviewers. We strongly encourage code deposition in a community repository (e.g. GitHub). See the Nature Research guidelines for submitting code & software for further information.

Data

Policy information about availability of data

All manuscripts must include a data availability statement. This statement should provide the following information, where applicable:

- Accession codes, unique identifiers, or web links for publicly available datasets
- A list of figures that have associated raw data
- A description of any restrictions on data availability

Source data and uncut gels for all blots used for generating the figures in this manuscript and in the Supplementary Information are provided with this paper.

Field-specific reporting

Please select the one below that is the best fit for your research. If you are not sure, read the appropriate sections before making your selection.

[x] Life sciences [ ] Behavioural & social sciences [ ] Ecological, evolutionary & environmental sciences

For a reference copy of the document with all sections, see nature.com/documents/nr-reporting-summary-flat.pdf

Life sciences study design

All studies must disclose on these points even when the disclosure is negative.

Sample size	No sample size calculation was performed. Sample size is always specified in every figure legend and experiments were generally repeated at least three times. Samples sizes are consistent with similar published studies. For mice experiments using nude mice, we expect to observe a difference rate >=30 between groups. To achieve statistical significance, we used 10 mice in each group, which gave a P value of 0.025 (CI: 95%) in a Pearson's Chi squared test.
Data exclusions	No data were excluded from the analysis.
Replication	Every experiment was replicated at least two to three independent experiments with similar results and the number of repeats is stated in individual figure legends.
Randomization	Mice were randomized into treatment groups. No other experimental grouping requiring randomization was performed.
Blinding	Mice tumor sizes were measured blindingly. Although investigators were not blinded to all other experiments, data collection was performed in an unbiased manner and the analysis was performed on the endpoints, which is not subject to investigators' bias.

Reporting for specific materials, systems and methods

We require information from authors about some types of materials, experimental systems and methods used in many studies. Here, indicate whether each material, system or method listed is relevant to your study. If you are not sure if a list item applies to your research, read the appropriate section before selecting a response.

Materials & experimental systems

n/a	Involved in the study	
[ ]	[x]	Antibodies
[ ]	[x]	Eukaryotic cell lines
[x]	[ ]	Palaeontology and archaeology
[ ]	[x]	Animals and other organisms
[x]	[ ]	Human research participants
[x]	[ ]	Clinical data
[x]	[ ]	Dual use research of concern

Methods

n/a	Involved in the study	
[x]	[ ]	ChIP-seq
[ ]	[x]	Flow cytometry
[x]	[ ]	MRI-based neuroimaging

Antibodies

Antibodies used	Western blotting:<br>Rabbit anti-S6K1 (1:1000; Abcam 32359)<br>Rabbit anti-pS6K-Thr389 (1:1000; CST 9205)<br>Rabbit anti-p4EBP1-Ser65 (1:1000; CST 9451)<br>Rabbit anti-4EBP1 (1:5000; CST 9644)<br>Rabbit anti-pan AKT (1:1000; CST 4691)<br>Rabbit anti-pAKT-Thr308 (1:1000; CST 2965)<br>Rabbit anti-AKT1 (1:1000; CST 2938)

Rabbit anti-pAKT substrate (1:1000; RXRXXpS*/T*) (CST 10001)
Rabbit anti-GAPDH (1:3000; CST 5174)
Mouse anti-flag (1:3000;Sigma F1804)
Mouse anti-flag (1:1000; Sigma A9594)
Rabbit anti-HA (1:1000; CST 3724)
Rabbit anti-HA (CST 3444)
Rabbit anti-GST (1:1000; CST 2625)
Mouse anti-Ub (1:300; Santa Cruz sc-8017)
Mouse anti-c-Myc (1:1000; Santa Cruz sc-40)
Mouse anti-RNF167 (1:400; Santa Cruz sc-515405)
Rabbit anti-RNF167 (1:400; Proteintech 24618-1-AP)
Mouse anti-β-tubulin (1:1000; Sigma 7B9)
Rabbit anti-CASTOR1 (1:1000; Chemipeptide)
Mouse anti-Rabbit IgG (1:2000; Light-Chain Specific) (CST 93702)
Rabbit anti-Mouse IgG (1:2000; Light Chain Specific) (CST 58802)
Goat anti-rabbit HRP conjugated IgG (1:3000; CST 7074)
Horse anti-mouse IgG HRP conjugated IgG (1:3000; CST 7076)
Goat anti-mouse IgG DyLight 800 (1:3000; Bio-Rad STAR117D800GA)
Goat anti-rabbit IgG StarBright Blue700 (1:3000; Bio-Rad 12004161)

Immunoprecipitation:
Mouse IgG agarose beads (1:250; Sigma A0919)
Mouse anti-Flag agarose beads (1:250; Sigma A2220)
Rabbit anti-HA agarose beads (1:250; Thermo 26182)
Rabbit anti-Myc agarose beads (1:250; Sigma A7470)
Rabbit anti-AKT agarose beads (1:250; CST 3653)
mouse IgG antibodies (1:250; Sigma A0919)

Validation

Rabbit anti-S6K1: validated by the manufacturer using HAP1 cell lysate and by our group in Hela, 293T, MCF7, T47D, HCC1569, HSAEC and HLBEC cells for immunoblotting application.
Rabbit anti-pS6K-Thr389: validated by the manufacturer using Hela, COS, C6 and 3T3 cell lysates and by our group in Hela, 293T, MCF7, T47D, HCC1569, HSAEC and HLBEC cells for immunoblotting application.
Rabbit anti-p4EBP1-Ser65: validated by the manufacturer using 293T cell lysates and by our group in Hela, 293T, MCF7, T47D, HCC1569, HSAEC and HLBEC cells for immunoblotting application.
Rabbit anti-4EBP1: validated by the manufacturer using Hela cell lysates and by our group in Hela, 293T, MCF7, T47D, HCC1569, HSAEC and HLBEC cells for immunoblotting application.
Rabbit anti-pan AKT: validated by the manufacturer using NIH/3T3, C6, COS and Hela cell lysates and by our group in 293T, MCF7, T47D, HCC1569 and HCC202 cells for immunoblotting application.
Rabbit anti-pAKT-Thr308: validated by the manufacturer using NIH/3T3 and Jurkat cell lysates and by our group in 293T, MCF7, T47D, HCC1569 and HCC202 cells for immunoblotting application.
Rabbit anti-AKT1: validated by the manufacturer using NIH/3T3, C6, COS-7, MEF and Hela cell lysates and by our group in MCF7, T47D and 293T cells for immunoblotting application.
Rabbit anti-pAKT substrate: validated by the manufacturer using MKN-45 and A-431 cell lysates and by our group in MM, KMM and 293T cells for immunoblotting application.
Rabbit anti-GAPDH: validated by the manufacturer using NIH/3T3, C6, COS-7 and Hela cell lysates and by our group in Hela, 293T, MCF7, T47D, HCC1569, HCC202, HSAEC and HLBEC cells for immunoblotting application.
Mouse anti-flag (Sigma F1804): validated by the manufacturer using MDCK canine kidney epithelial cells and by our group in 293T, MCF7 and T47D cells for immunoblotting application.
Mouse anti-flag (Sigma A9594): validated by the manufacturer using NIH/3T3, C6, COS-7 and Hela cells and by our group in 293T cells for immunoblotting application.
Rabbit anti-HA (CST 3724): validated by the manufacturer using COS, 293T and Hela cells and by our group in MCF7, T47D and 293T cells for immunoblotting application.
Rabbit anti-HA (CST 3444): validated by the manufacturer using COS cells and by our group in 293T cells for immunoblotting application.
Rabbit anti-GST: validated by the manufacturer using COS-7 Hela cell lysates and by our group in in vitro kinase assay to detect recombinant GST-CASTOR1 and GST-AKT1 proteins.
Mouse anti-Ub: validated by the manufacturer using NIH/3T3 and Jurkat cells and by our group in 293T cells for immunoblotting application.
Mouse anti-c-Myc: validated by the manufacturer using Jurkat and K-562 cell lysates and by our group in 293T cells for immunoblotting application.
Mouse anti-RNF167: validated by the manufacturer using COLO 205, Caki-1, JAR, TT, PC-12, F9 cell lysates and rat kidney tissue extract. Our group validated this antibody in 293T cells for immunoblotting application by knocking down this gene using siRNA or overexpressing this protein.
Rabbit anti-RNF167: validated by the manufacturer using SH-SY5Y cell lysates, mouse testis and brain tissues and by our group in MCF7, T47D, HCC1569 and HCC202 cells for immunoblotting application.
Mouse anti-β-tubulin (1:1000; Sigma 7B9): validated by the manufacturer using 293T and Hela cell lysates and by our group in 293T, MCF7 and T47D cells for immunoblotting application.
Rabbit anti-CASTOR1 (1:1000; Chemipeptide): validated in the published paper using rat MM and KMM cell lysates for immunoblotting application. Our group validated this antibody in 293T, MCF7, T47D, HCC1569 HSAEC, HLBEC and HCC202 cells for immunoblotting application by knocking down this gene using siRNA or overexpressing this protein.
Mouse anti-Flag agarose beads: validated in numerous published papers and by our group in 293T cells for immunoprecipation

application.
Rabbit anti-HA agarose beads: validated by the manufacturer using HA-tagged GFP expressed from Pontellina plumata in vitro and purified by incubation with this anti-HA agarose. Our group validated this antibody in 293T cells for immunoprecipitation assay.
Rabbit anti-Myc agarose beads: validated in numerous published papers and by our group in 293T cells for immunoprecipitation application.
Rabbit anti-AKT agarose beads: validated by the manufacturer using Jurkat cell lysates and by our group in 293T cells for immunoprecipitation application.

Eukaryotic cell lines

Policy information about cell lines

Cell line source(s)	293T cells were obtained from ATCC (CRL-3216). Hela, MCF7, T47D, HCC1569 and HCC202 cells were obtained from Dr. Xiaosong Wang at the University of Pittsburgh. HSAEC (FC-0016) and HLBEC (FC-0054) cells were purchased from Lifeline.
Authentication	The cell lines were not authenticated.
Mycoplasma contamination	All cell lines were validated to be free of mycoplasma contamination.
Commonly misidentified lines (See ICLAC register)	No commonly misidentified cell lines were used.

Animals and other organisms

Policy information about studies involving animals; ARRIVE guidelines recommended for reporting animal research

Laboratory animals	Female athymic Nude-Foxn1nu mice of 5-6 weeks old were purchased from Envigen. Mice were raised under 12 h light/dark cycle and with standard diet at the University of Pittsburgh. Mice were housed under 65-75°F (~18-23°C) with 40%~60% humidity. Mice were housed in plastics cages (no more than five animals per cage to minimize aggressiveness).
Wild animals	No wild animals were used in the study.
Field-collected samples	No field collected samples were used in the study.
Ethics oversight	Animal welfare was ensured, and experimental procedures were performed in strict accordance with animal welfare and other related ethical regulations, and the procedures were approved by the Institutional Animal Care Committee of the University of Pittsburgh (IACUC, Protocol #: 18073052).

Note that full information on the approval of the study protocol must also be provided in the manuscript.

Flow Cytometry

Plots

Confirm that:

- [x] The axis labels state the marker and fluorochrome used (e.g. CD4-FITC).
- [x] The axis scales are clearly visible. Include numbers along axes only for bottom left plot of group (a 'group' is an analysis of identical markers).
- [ ] All plots are contour plots with outliers or pseudocolor plots.
- [x] A numerical value for number of cells or percentage (with statistics) is provided.

Methodology

Sample preparation	For BrdU incorporation, MCF7 or HCC1569 cells were pulsed with 10uM BrdU (Sigma B5002) for 2 h, and then fixed with 70% ethanol, permealized with 2 M hydrochloric acid and stained with an anti-BrdU monoclonal antibody (Thermo B35129). The apoptotic cells of MCF7 or HCC1569 were detected by co-staining with DAPI (Sigma D9542) and PE-Cy7 Annexin V Apoptosis Detection kit (eBioscience 88810374). Flow cytometry was performed in a BD LSRFortessa system (BD Biosciences) and the analysis was done with FlowJo.
Instrument	BD LSRFortessa system (BD Biosciences).
Software	Flow cytometry data was collected by BD LSRFortessa system and analyzed by Flowjo vX.0.7
Cell population abundance	A total of 100,000 cells was examined by BD LSRFortessa system.
Gating strategy	For Brdu incorporation assay, the DNA of MCF7 or HCC1569 cells stained with propidium iodide that are from 2N to 4N were gated for analysis. The de novo synthesis of DNA that are 2N to 4N is quantified by the intensity of Brdu. For apoptosis assay, cell debris are ruled out by gating with the FFS/SSC . The remaining cells are stained with both DAPI and annexin-V for apoptotic and dead cells.

- [x] Tick this box to confirm that a figure exemplifying the gating strategy is provided in the Supplementary Information.
